# Silastic Joint Arthroplasty as a Joint-Preserving Alternative for End-Stage Hallux Rigidus: Outcomes From 112 First Metatarsophalangeal Joint Arthroplasties

**DOI:** 10.7759/cureus.46561

**Published:** 2023-10-06

**Authors:** Mohit Sethi, Natalie Limaye, Elizabeth Alderton, Rajiv Limaye, Ameet Kulkarni

**Affiliations:** 1 Orthopaedics and Trauma, North Tees and Hartlepool NHS Foundation Trust, Stockton-on-Tees, GBR

**Keywords:** swansons implant, nice, silastic implants, first mtpj, hallux rigidus

## Abstract

Aim

Osteoarthritis of the first metatarsophalangeal joint (MTPJ) is a common forefoot problem affecting patients in later years. It leads to pain, gait problems, and difficulty with activities of daily living. Treatment is controversial and varies according to patient symptoms and surgeon preference. Arthrodesis remains the gold standard but it has its own complications. It is associated with adjacent joint arthritis and transfer metatarsalgia. The aim of this study was to analyze the outcome of double-stemmed silastic joint arthroplasty (Wright-Medical, Memphis, TN) for end-stage hallux rigidus.

Methods

This retrospective analysis included 117 consecutive first MTPJ silastic arthroplasties done between January 2016 and February 2023 for end-stage hallux rigidus. There were 77 females and 40 males with a mean age of 65 years (46-82 years). Radiological and clinical assessments were performed, and patient-reported outcome measure data (PROMS) and visual analogue scale (VAS) scores were collected pre- and post-operatively.

Results

Findings showed 99.1% survivorship following a silastic joint arthroplasty with a mean follow-up of four years (six months to seven years). The MOXFQ (Manchester Oxford Foot Questionnaire) score improved from a mean of 81 (59.8-100) to 13 (0-57). The mean VAS scores improved from 7.2 (5-10) to 1.5 (0-7) postoperatively. Five patients were lost to follow-up. Two patients developed deep infection and one required revision. The other patient with infection was lost to follow-up. In total 10 patients (8.9%) developed complications, out of which eight patients responded to simple treatments.

Conclusion

Results have shown good to excellent outcomes following a silastic arthroplasty of the first MTPJ for the treatment of end-stage hallux rigidus. The survivorship at a mean follow-up of four years was 99.1% and the patient satisfaction rate was 90.1%. As historically reported, we did not see any soft tissue reaction or progressive osteolysis in any of our patients. It provides comparable and predictable outcomes to joint fusion for end-stage arthritis.

## Introduction

Osteoarthritis of the first metatarsophalangeal joint (MTPJ) is a common condition that forms a bulk of orthopaedic foot and ankle practice. It affects between 35% and 65% of adults older than 65 years [1]. It is a degenerative condition that can result from trauma, footwear problems and biomechanical instability [2]. Hallux rigidus can lead to difficulty in activities of daily living and an altered gait pattern. Non-operative measures form the mainstay of the treatment which include altered footwear, orthotics and activity modification. Surgical treatment is controversial and is offered to patients with end-stage arthritis. There is no agreed consensus on which procedure gives better long-term results. The traditional options for operative management of end-stage arthritis include excision arthroplasty, implant arthroplasty and arthrodesis. Excision arthroplasty has gradually become less popular with a limited value in current practice [3-5]. Arthrodesis remains the gold standard for treating end-stage arthritis due to predictable results and surgeon familiarity and is associated with high non-union rates (2-10%) and the need for implant removal (10-14%) [2,6]. Arthroplasty is a good alternative to arthrodesis as it preserves joint space and helps to maintain the gait pattern. There is no associated risk of non-union, malunion or transfer metatarsalgia. Joint replacement for the first MTP joint can be done with silastic or metallic implants. The initial results with silastic implants were not promising, which led to its decreased use. A recent study has shown that arthroplasty of the first MTP joint produces similar results and satisfaction rates as compared to arthrodesis [7]. The National Institute of Health and Care Excellence suggests that there is adequate evidence to support the use of silastic joint arthroplasty. It recommends that patients should be appropriately consented and correct arrangements should be made for clinical governance.

Swanson first introduced silastic implants in the mid-1960s to improve the results of Keller’s arthroplasty. They were single-stemmed implants designed to replicate the function of the first MTP joint. These implants were withdrawn from the market within four years due to high failure rates [8]. Complications included synovitis, granuloma formation, osteolysis and implant migration. Subsequently, double-stemmed implants were introduced which gave more favourable results. Current third-generation silastic implants are now in use, and give more predictable results. Despite low complication rates and improved results with current implants, it remains a controversial surgical option and is rarely used in clinical practice.

The primary aim of this study was to assess the functional outcome and longevity of Swanson’s silastic joint arthroplasty. Results were based on patient-related outcome measures (PROMS), radiological outcomes and patient satisfaction.

## Materials and methods

A retrospective analysis was conducted with 117 consecutive first MTPJ silastic arthroplasties done between January 2016 and February 2023 for end-stage hallux rigidus. All the surgeries were performed by a single surgeon across three different hospitals. Data was collected from the hospital database and PROMS was assessed pre- and post-operatively. There were 77 females and 40 males and the mean age was 65 years at the time of surgery (46-82 years).

Operative procedure

**Figure 1:**
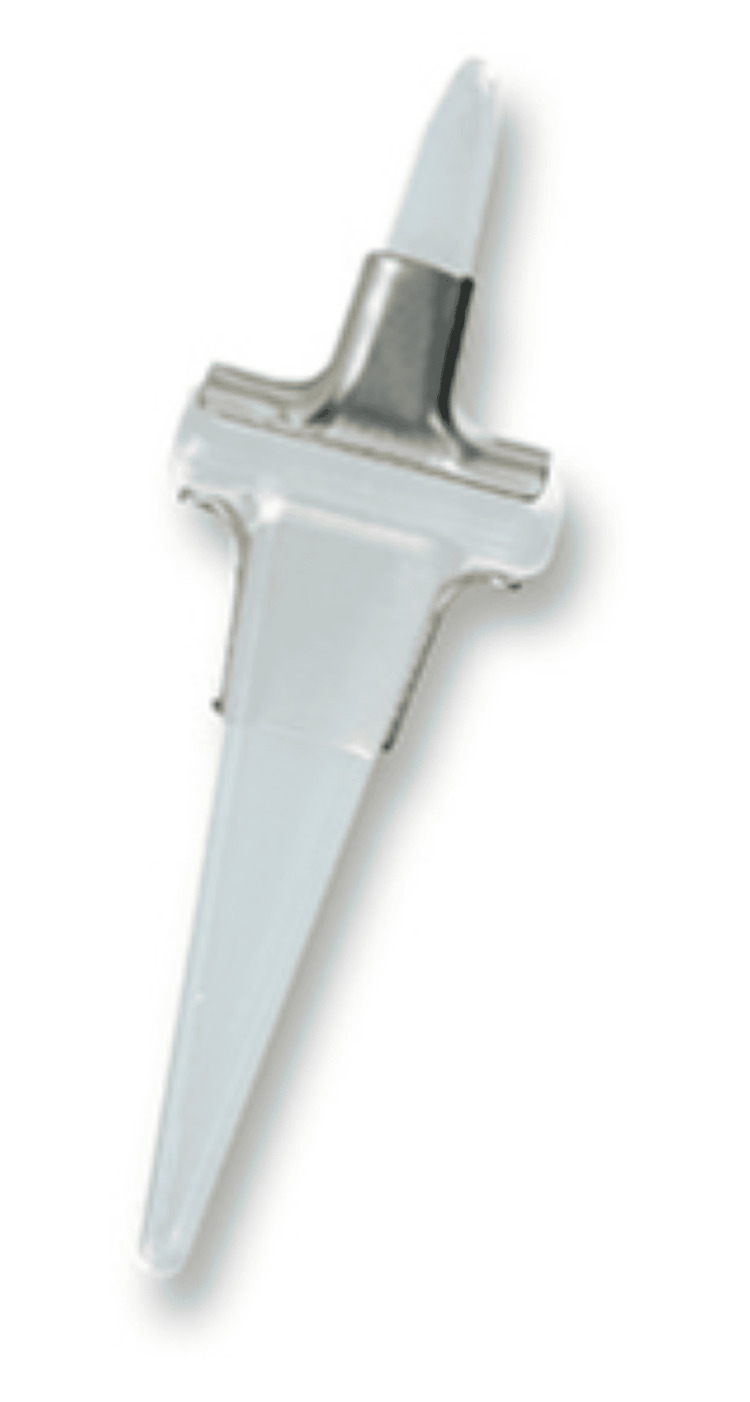
Double-stemmed silastic joint (Wright-Medical, Memphis, Tennessee, USA)

Surgeries were performed as day cases, with patients consented to the clinic prior to surgery. Symptoms and consent forms were verified on the day of surgery. All the surgeries were performed in an orthopaedic theatre with laminar flow. A pneumatic thigh tourniquet was used during surgery and all patients received a single preoperative dose of antibiotic and an ankle block for pain relief postoperatively. The standard dorsomedial approach was used for all patients. After exposing and dislocating the joint, proximal and distal bone cuts were made. A proximal cut was made just distal to the metaphyseal flare at the longest diameter of the metaphyseal head. A portion of the base of the distal base of the proximal phalanx was removed to provide wider joint space and the canal was broached on either side. After determining the correct size, the silastic implant was inserted. No grommets were used in these cases. Excess osteophytes were removed and sharp edges were trimmed. Capsular plication was done, with Vicryl (Ethicon, Inc., Somerville, New Jersey) and nylon used for wound closure purposes. A single post-operative X-ray was done using an image intensifier to confirm the positioning of the implant. The post-operative regime included heel weight bearing for two weeks and removal of sutures at two weeks. Thereafter full weight bearing was commenced as tolerated. All the patients received physiotherapy in the form of gait training, proprioception training and range of motion exercises. Patients were followed up at six weeks, three months, six months and one year. Pre- and post-operative PROMS were available for 112 patients. Patient satisfaction was measured by a questionnaire and patients were asked if they were happy with their joint replacement. Figure 1 shows the Silastic Implant used for the first MTPJ arthroplasty.

Radiological assessment

All the patients were assessed pre- and post-operatively with weight-bearing anteroposterior (AP) and lateral radiographs. The alignment and severity of arthritis were assessed before and after the surgery. All patients had advanced arthritis with bone-on-bone changes. Osteolysis was defined as lucency of more than 2mm. Revision due to any cause was regarded as a failure or primary procedure and an endpoint.

Functional outcome

All the patients were assessed according to PROMS that were collected pre- and post-operatively. MOXFQ (Manchester Oxford Foot Questionnaire) and VAS (visual analogue score) were used to quantify pain. MOXFQ is a foot and ankle-based score that assesses patients across three different subscales - walking/standing, pain and social interaction. The score is converted to a scale between 0 and 100 where 100 indicates the worst outcome. VAS assesses pre- and post-operative pain where 0 is no pain and 10 is severe pain.

## Results

**Figure 2:**
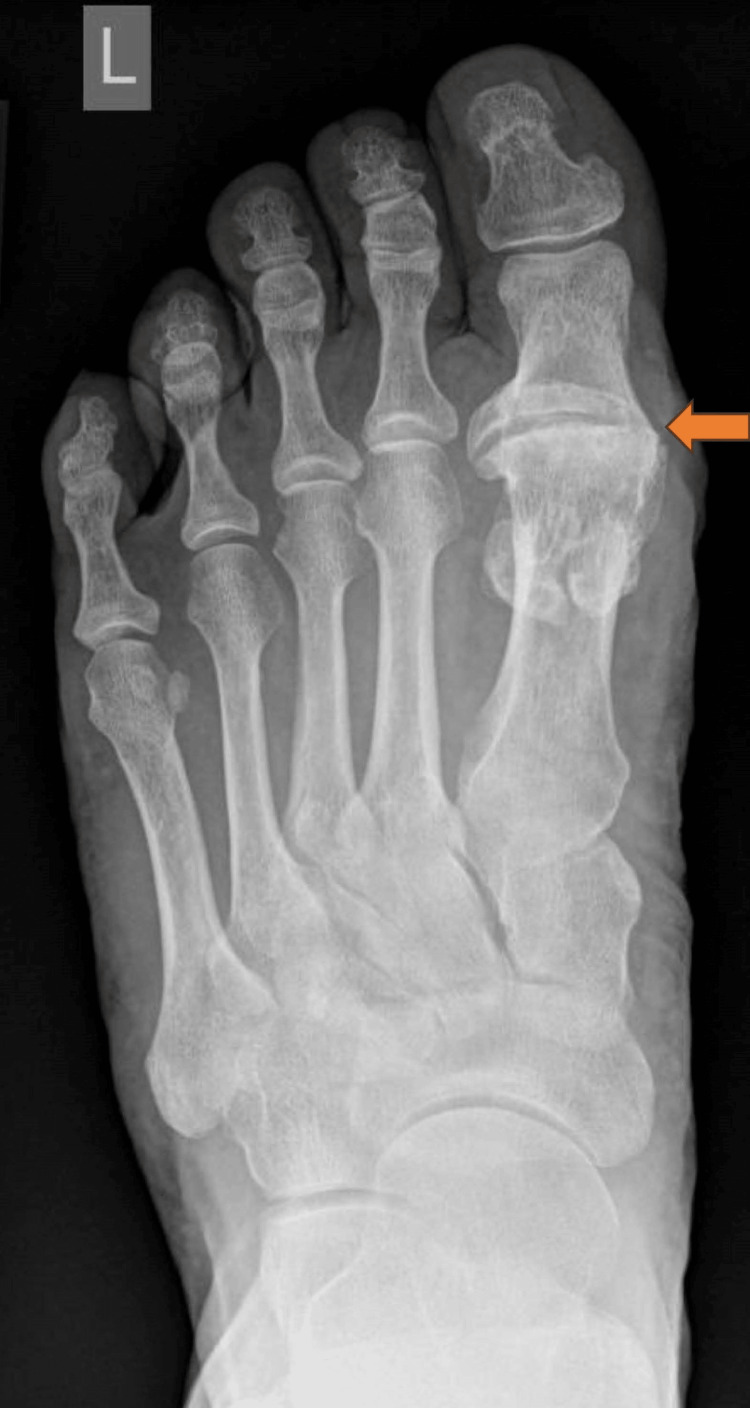
Pre-operative Radiograph Showing First Metatarsophalangeal Joint Arthritis

**Figure 3:**
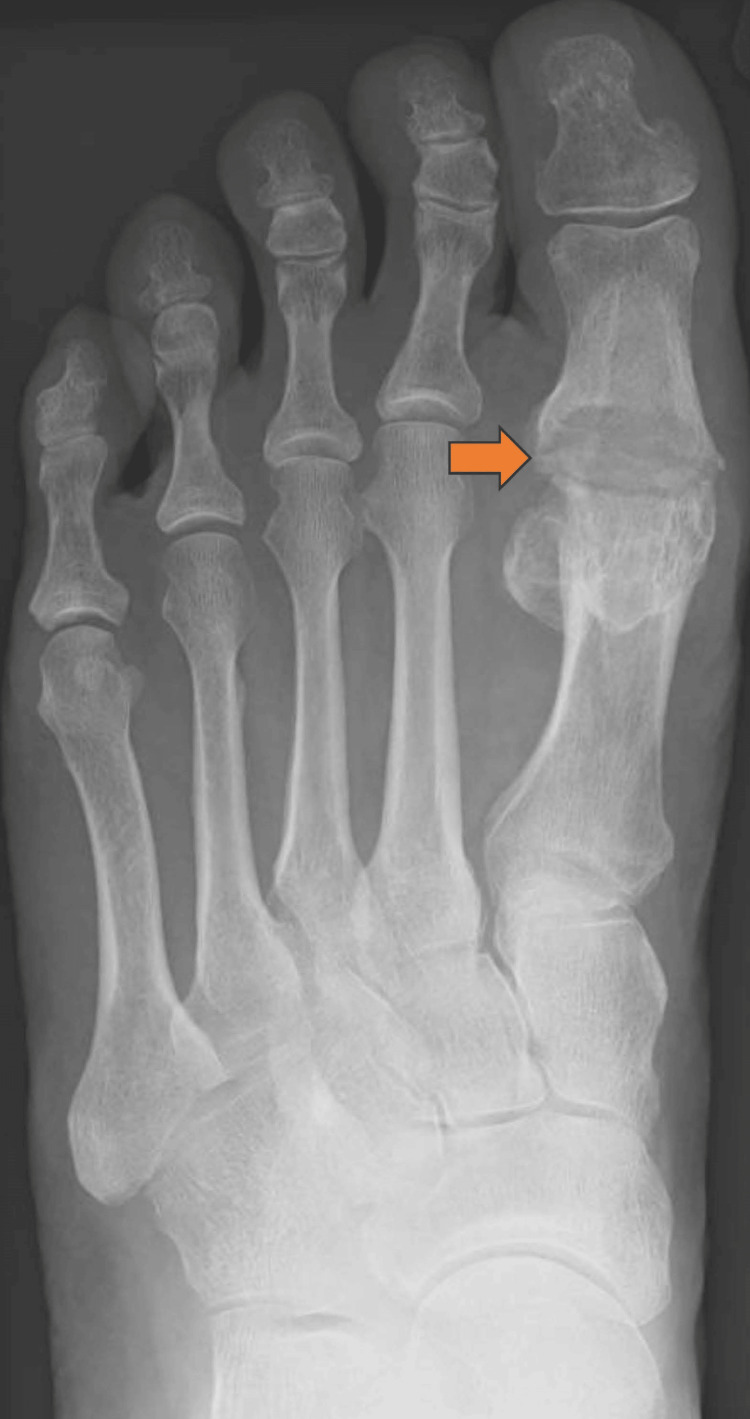
Post-operative Radiograph Showing Silastic First Metatarsophalangeal Joint Replacement

There were a total of 117 patients out of which 112 patients were available for assessment. Five patients were lost to follow-up. Two patients developed a deep infection and one required surgical intervention. The patient had a two-stage procedure. In the first stage, we took the implant out, debrided the canal and started antibiotics as per sensitivity. Subsequently, 12 weeks down the line we fused the joint. The infection settled down and the patient was symptom-free at six months. The second patient with infection was unfortunately lost to follow-up. One patient developed asymptomatic osteolysis that did not require any treatment. The patient is still under follow-up and we are monitoring patient symptoms and implant survivorship. Table 1 shows complications of silastic joint replacement in 112 patients. Figures 2, 3 show pre- and post-operative radiographs following the successful first MTPJ arthroplasty.

**Table 1:** Complications of First Metatarsophalangeal Silastic Joint Replacement in 112 Patients CRPS: complex regional pain syndrome

Complication	% (n)	note
Stiffness	1.9 (2)	Responded to physiotherapy
Lateral metatarsalgia	2.6 (3)	Treated with orthotics and footwear modification
Superficial infection	0.9 (1)	Responded to oral antibiotics
CRPS	0.9 (1)	Settled at one year post-operatively
Wound Dehiscence	0.9 (1)	Healed with dressings
Deep Infection	0.9 (1)	Required revision surgery (debridement, antibiotics and arthrodesis)
Asymtomatic Osteolysis	0.9 (1)	Under observation

Two patients complained of stiffness which responded to physiotherapy. One patient developed complex regional pain syndrome (CRPS) which settled at one year post-operatively. One patient had a superficial infection in the form of a suture abscess which responded to oral antibiotics. One patient had a wound breakdown which settled with dressings and antibiotics. There were no intraoperative phalangeal fractures. We have not seen any implant fractures so far in our series of patients. No patients developed deep vein thrombosis (DVT). Three patients had transfer metatarsalgia which settled down with orthotics and footwear modification. In total 10 patients (8.9%) developed complications, eight of which responded to simple treatments. The overall survivorship of the silastic implant in our study was 99.1%.

**Figure 4:**
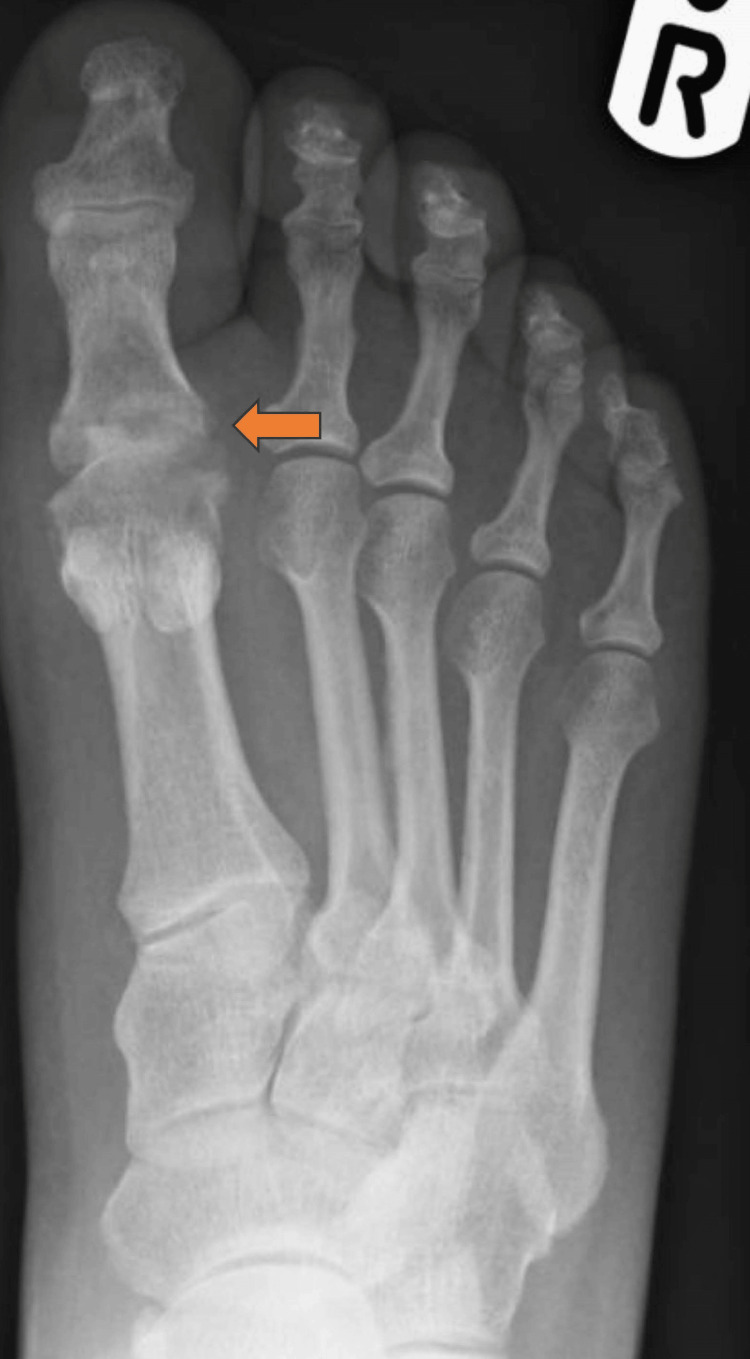
Radiograph Showing Infected First Metatarsophalangeal Joint Replacement

**Figure 5:**
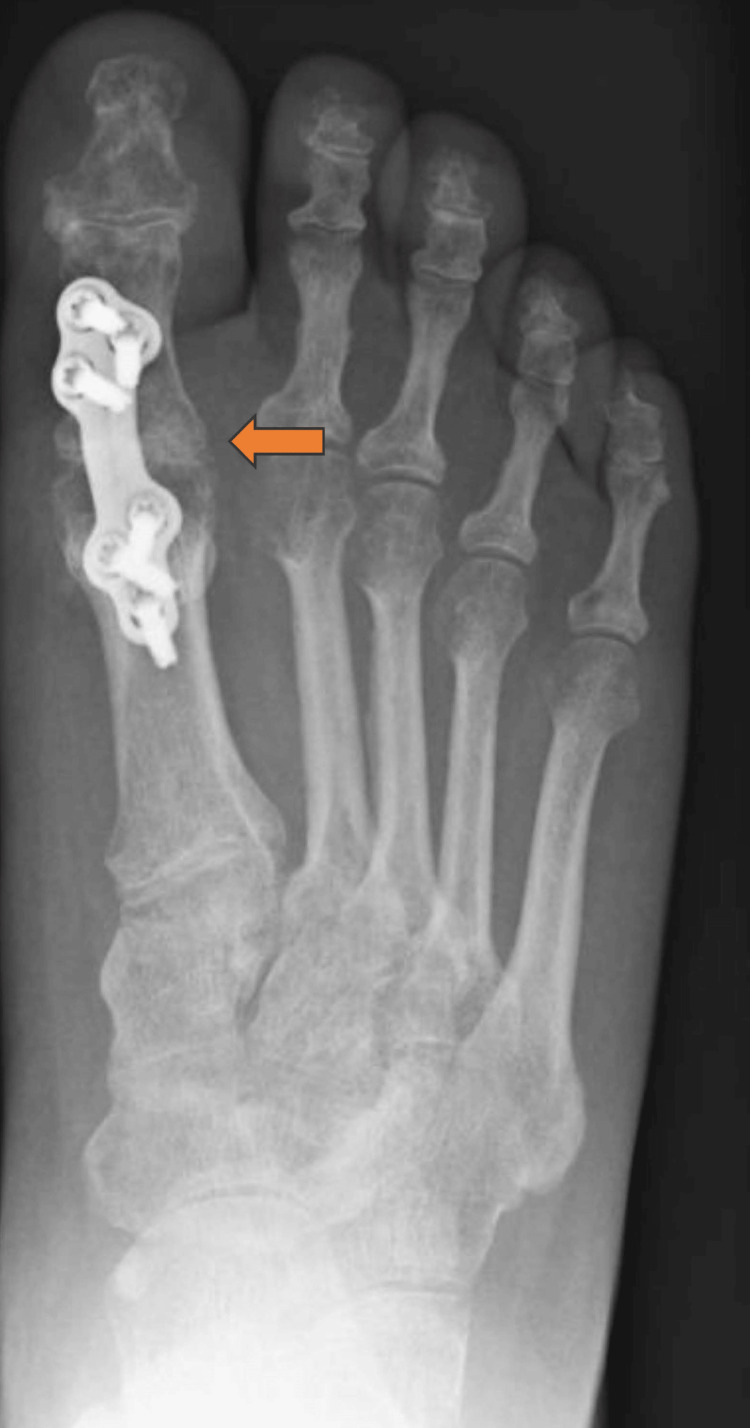
Radiograph Following Debridement and Arthrodesis at 12 weeks

Figure 4 shows a radiograph of the infected first MTPJ. Figure 5 shows a radiograph following debridement and arthrodesis at 12 weeks.

There was a significant improvement in MOXFQ as well as VAS scores postoperatively at the time of the last follow-up. MOXFQ score improved from a mean of 81 (59.8-100) to 13 (0-57). The mean VAS scores improved from 7.2 (5-10) to 1.5 (0-7) post-operatively.

Overall, 101/112 patients were satisfied with the outcome of surgery, giving a satisfaction rate of 90.1%. Patients were retrospectively assessed up to their last follow-up, which revealed no radiological abnormalities in 110/112 (98.2%) of patients. One patient had some asymptomatic osteolysis around the implant, which did not require any treatment.

## Discussion

At a mean follow-up of four years, a survivorship rate of 99.1% was reported along with a 90.1% patient satisfaction rate when using a double-stemmed silastic implant. There was improvement in both MOXFQ and VAS scores indicating a favourable outcome. No symptomatic loosening, synovitis or progressive cyst formations were seen. No postoperative hallux valgus deformity was seen in any of the patients, as historically there have been reports of patients developing hallux valgus after silastic joint arthroplasty [8-12]. Progressive cyst formation is a known complication with first and second-generation implants but we did not see any such complication in our study [13-17].

Recently a study conducted by Clough et al. [15] reported comparable results in 108 patients, reporting a 97.2% survivorship rate. They also included a cost analysis to determine the cost-effectiveness within NHS (National Health Service, UK) which found that cost is favourable (265 GBP) when compared to other implants including screw and plate fixation (666-951 GBP). Keurs et al. [16] in a similar study reported a 94.9% survivorship rate in their series of 48 patients at a mean follow-up of nine years. Morgan et al. [14] reported a survivorship of 96.9% in their series of 97 patients at a mean follow-up of 8.5 years. Van Duijvenbode et al. [18] reported the results of 36 patients at a mean follow-up of 19 years. They reported good to excellent outcomes with a 4% revision rate, one being a revision to a further silastic arthroplasty at nine years, with two patients requiring revision surgery to Keller’s procedure. In a recent study, Fieschi et al. [19] reported excellent results with no revision rates at a mean follow-up of seven years. They used a Primus silastic implant (Integra, Plainsboro, New Jersey, USA) that is structurally different to the Swansons implant used in this current study. They reported an 18.6% osteolysis rate in their series. They also reported a bone resorption rate of 53% when grommets were used.

One of the other implants used for hallux rigidus end-stage arthroplasty includes Bio Pro (BioPro, Port Huron, Michigan, USA). Townley et al. [20] reported higher than normal revision rates in the younger population and a survival rate of 85.6 % at 5.4 years. It is a resurfacing metallic implant for the proximal phalanx. Historically there have been reports of plantar cut-out and transfer metatarsalgia with this implant.

Another implant used for end-stage hallux rigidus is MOJE (Sovereign-Medical, Stansted, UK). It is a ceramic implant which relies on a press fit on either side. It was first introduced as a screw fit but was discontinued due to high rate of subsidence and fracture [21,22]. It has been associated with fair to poor clinical outcomes in mid- to long-term studies. Abruthnot et al. [23] reported early outcomes at a mean follow-up of 1.8 years which were encouraging, reporting a 97.5% satisfaction rate. However, concerns were raised when Dawson-Bowling et al. [24] also reported a 25% revision rate at a mean follow-up of 4.8 years in their series of 31 implants.

The Cartiva (Cartiva, Alpharetta, Georgia, USA) is an implant that is used in moderate arthritis and has different indications when compared to the silastic implant. It is made up of polyvinyl alcohol hydrogen and aims to replacing the metatarsal head. Baumhauer et al. [25] in their series of 152 patients reported improved pain but also reported a revision rate of 9.2% at two years. Mid-term results in the same series showed a 16.8% revision rate, including conversion to arthrodesis at five years. Casseneli et al. [26] reported a 20% revision rate with an 8% conversion rate to arthrodesis at a mean follow-up of 18.5 months. They reported that 38% of patients were ‘unsatisfied’ or ‘very unsatisfied’ with surgical outcomes. The initial results with Cartiva implant (Cartiva, Alpharetta, GA) were very promising and showed early and faster recovery. Unfortunately due to a higher than normal revision rate in mid-term, the debate is still on with regards to the survivorship of the implant.

Roto-glide (Implants-International, Stockton-on-Tees, UK), Toefit-Plus (Smith and Nephew, Watford, UK), Metis (Integra, Plainsboro, New Jersey, USA), and Bio-Action (Osteomed, Addison, Texas, USA) and three-component implants which have shown good early results [27-30]. In our earlier series (Karpe et al. [31]), we reported a revision rate of 8.8% in 28 patients at a mean follow-up of 2.3 years with a Roto-glide implant. A similar study done by Titchner et al. [32] reported results of 86 Toefit-Plus implants. They reported a high revision rate of 24% with a high refracture rate of 9.3% at a mean follow-up of 33 months. Sinha et al. [33] reported the results of 15 Bio-Action implants at a mean follow-up of 5.1 years. They reported a loosening rate of 93.3% and 86.6% for the phalangeal and metatarsal side respectively.

There were some limitations in this study which can be addressed in future studies. Data was collected retrospectively and assessed for outcomes. A prospective RCT in future would give more accurate results. Pre- and post-operative range of motion (ROM) was not assessed, and would have been a valuable addition to the outcome analysis. We plan to publish the 10-year results in future, to assess the long-term survival and patient satisfaction rates.

## Conclusions

We suggest that the silastic implant is a good device to treat end-stage hallux rigidus, although strict indications should be followed. It is a silastic hinge spacer and not a device to correct deformity, hence its use should be avoided in patients with hallux valgus/varus deformity. It relies on the viscoelastic properties of the silastic implant to align itself in the long axis of the joint. It is not recommended to use silastic implants in osteoporotic bones or in patients with rheumatoid/inflammatory arthritis due to the risk of fracture, osteolysis and cyst formation.

It is understood that the previously reported results with this implant were not favourable however recent studies from Wrightington show good to excellent outcomes with silastic implants, as does this current study. Findings concluded good pain reduction, excellent PROMS and patient satisfaction with minimal complications. Most of the studies done previously were with single-stemmed implants, however, with current-generation implants, these complications are seen less frequently. Options for failed silastic joint arthroplasty include a revision to a silastic device or fusion with a bone graft. Overall, silastic joint arthroplasty for end-stage hallux rigidus with double-stemmed implants provides good to excellent outcomes in mid-term survival.
